# Evaluation of models to predict BRCA germline mutations

**DOI:** 10.1038/sj.bjc.6603358

**Published:** 2006-10-03

**Authors:** H H Kang, R Williams, J Leary, kConFab Investigators, C Ringland, J Kirk, R Ward

**Affiliations:** 1 Department of Medical Oncology, St Vincent's Hospital, Sydney, New South Wales, Australia; 2 Familial Cancer Service, Westmead Institute for Cancer Research at Westmead Millennium Institute, University of Sydney, Westmead, Sydney 2052, Australia; 3 Kathleen Cuningham Consortium for Research into Familial Breast Cancer, Peter MacCallum Cancer Institute, St Andrews Place, East Melbourne, Victoria 3002, Australia; 4 School of Medical Sciences, University of NSW, Sydney 2052, Australia; 5 St Vincent's Clinical School, University of NSW, Sydney 2052, Australia

**Keywords:** breast cancer, models, BRCA, risk, mutations

## Abstract

The selection of candidates for BRCA germline mutation testing is an important clinical issue yet it remains a significant challenge. A number of risk prediction models have been developed to assist in pretest counselling. We have evaluated the performance and the inter-rater reliability of four of these models (BRCAPRO, Manchester, Penn and the Myriad-Frank). The four risk assessment models were applied to 380 pedigrees of families who had undergone BRCA1/2 mutation analysis. Sensitivity, specificity, positive and negative predictive values, likelihood ratios and area under the receiver operator characteristic (ROC) curve were calculated for each model. Using a greater than 10% probability threshold, the likelihood that a BRCA test result was positive in a mutation carrier compared to the likelihood that the same result would be expected in an individual without a BRCA mutation was 2.10 (95% confidence interval (CI) 1.66–2.67) for Penn, 1.74 (95% CI 1.48–2.04) for Myriad, 1.35 (95% CI 1.19–1.53) for Manchester and 1.68 (95% CI 1.39–2.03) for BRCAPRO. Application of these models, therefore, did not rule in BRCA mutation carrier status. Similar trends were observed for separate BRCA1/2 performance measures except BRCA2 assessment in the Penn model where the positive likelihood ratio was 5.93. The area under the ROC curve for each model was close to 0.75. In conclusion, the four models had very little impact on the pre-test probability of disease; there were significant clinical barriers to using some models and risk estimates varied between experts. Use of models for predicting BRCA mutation status is not currently justified for populations such as that evaluated in the current study.

Germline mutations in BRCA1 and BRCA2 confer an estimated 65 and 45% cumulative lifetime risk of developing breast cancer, and an ovarian cancer risk of 39 and 11%, respectively (Antoniou *et al*, 2003). Identification of these individuals before they present with cancer is important since prophylactic surgery can reduce morbidity and mortality in these individuals (Struewing *et al*, 1995; Rebbeck *et al*, 1999; Hartmann *et al*, 1999; Hartmann *et al*, 2001; Meijers-Heijboer *et al*, 2001; Kauff *et al*, 2002; Rebbeck *et al*, 2004). Unfortunately, genetic testing is expensive, family history of breast cancer is common and BRCA mutations are rare (Ford *et al*, 1994; Peto *et al*, 1999; Antoniou *et al*, 2002). A triage tool to identify those families most likely to benefit from germline testing would, therefore, be very useful. Like most countries (summarized by Nelson *et al*, 2005), Australia has developed guidelines (NBCC Genetics Working Group, 2000) for the referral of individuals to a family cancer clinic for counselling and further evaluation. Under these guidelines, full BRCA1/2 mutation screening is usually restricted to individuals affected by breast or epithelial ovarian cancer, and whose family history as a whole satisfies a ‘potentially high-risk category’ (NBCC Genetics Working Group, 2000; Nelson *et al*, 2005). The prevalence of germline BRCA mutations in such high-risk families is estimated at 3.4–15.5% (Frank *et al*, 2002; Huang *et al*, 2002; Antoniou *et al*, 2003; Myriad, 2004; Nelson *et al*, 2005). These low figures have led to the development of models that can more accurately assess the pre-test probability of identifying a BRCA1/2 germline mutation (Couch *et al*, 1997; Parmigiani *et al*, 1998; Berry *et al*, 2002; Frank *et al*, 2002; Evans *et al*, 2004). Mendelian models like BRCAPRO (Parmigiani *et al*, 1998; Berry *et al*, 2002) evaluate the probability that an individual is a gene mutation carrier, while other models such as Penn (Couch *et al*, 1997), Manchester (Evans *et al*, 2004) and Frank-Myriad (Frank *et al*, 2002) determine the likelihood of identifying a mutation on the basis of known family history. Although the performance of some of these models has been previously examined, it is perhaps telling that no single model has been universally adopted. Previous model validation studies have considered only a subset of the available models, have not compared the results with germline testing, and have not considered the barriers to the use of models in clinical practice (Berry *et al*, 2002; Euhus *et al*, 2002; de la Hoya *et al*, 2003; Marroni *et al*, 2004; James *et al*, 2006; Barcenas *et al*, 2006).

This study sought to compare the ability of four models (BRCAPRO, Manchester, Penn and the Myriad-Frank) to determine the likelihood of finding a BRCA gene mutation in at-risk individuals using a large group of patients who had undergone germline testing.

## METHODS

### Patient cohort

Family cancer clinics at St Vincent’s and Westmead Hospitals, Sydney, provided pedigrees of families who had undergone BRCA1/2 mutation analysis in the period 1998–2004. BRCA1/2 testing was performed using the DNA of an affected individual, usually the youngest living available affected person, or an obligate gene mutation carrier. For the purposes of the study, this individual was defined as the proband. Families in which no affected individuals were available for BRCA1/2 testing were not included in the study. To be eligible for germline testing, at least one unaffected family member must have had a life time risk of breast cancer of 1 : 4 or greater as defined by the Australian National Breast Cancer (NBCC) guidelines (NBCC Genetics Working Group, 2000). This included individuals with at least two first- or second-degree relatives on one side of the family diagnosed with breast or ovarian cancer, together with additional features on the same side of the family. These features included an additional relative with breast or ovarian cancer; breast cancer diagnosed before the age of 40 years, ovarian cancer before 50 years, bilateral breast cancer, breast and ovarian cancer in the same woman, Jewish ancestry or breast cancer in a male relative.

Each pedigree was de-identified, with only the sex, birthday, date of death, type of cancer and age at diagnosis retained for the analysis. Not all cancers could be verified because of the requirement for client consent in Australia. Pedigrees were viewed with Progeny software v5.4.05 (Progeny Software, LLC, South Bend, IN, USA). Furthermore, families of Ashkenazi Jewish ancestry were not included in this study. This study was approved by the St Vincents Hospital Human Research Ethics Committee.

### Use of models to identify mutation carriers

Each of the four risk assessment models (Manchester (Evans *et al*, 2004; Evans *et al*, 2005), Myriad (Frank *et al*, 2002), Penn (Couch *et al*, 1997) and BRCAPRO (Parmigiani *et al*, 1998; Berry *et al*, 2002)) were applied to each pedigree by two investigators (HK and RWi). All models except Myriad allow calculations for combined BRCA mutation status, as well as BRCA1 and BRCA2 independently. Risk assessments were based on the proband, and maternal or paternal inheritance was assessed using the Progeny program (Progeny Software, LLC, South Bend, IN, USA). If the potential carrier side was not obvious, the assessments were conducted for both paternal and maternal relatives and the highest score was included in the analysis. As data on some pedigrees was ambiguous or incomplete, prespecified rules were used for the entry of information into each model (Table 1). Determination of mutation likelihood using the Manchester model was performed by assigning a score on the basis of malignancy type and age of diagnosis (Evans *et al*, 2004). Scores obtained do not equate directly to probabilities; however, the authors suggest that a cutoff score of 10 points for BRCA1 and BRCA2 separately, or a combined score of 15 points correspond to an approximate mutation probability of 10% (Evans *et al*, 2004; Evans *et al*, 2005). The Myriad model was applied using on-line tables (Myriad, 2004) and an on-line questionnaire was used to obtain independent probabilities for BRCA1 and BRCA2 mutations using the Penn model (University of Pennsylvania Abramson Cancer Center, 2005). The latter model requires the presence of breast cancer in the family lineage and thus six families with only ovarian cancer could not be analysed. The overall chance of a mutation in either BRCA1 or BRCA2 was calculated as the probability of mutation in BRCA1 plus the probability of mutation in BRCA2 assuming there was no mutation in BRCA1. For the BRCAPRO model, carrier probabilities were calculated by entering information on the proband's first- and second-degree relatives into CancerGene software (CaGene version 3.3, supplied by Assistant Professor D Euhus, UT Southwestern Medical Center at Dallas, Texas, USA).

### Mutation screening for BRCA1 and BRCA2

The extent to which BRCA1/2 were screened reflects the availability of testing methodologies over the study period. Full DNA sequence analysis of BRCA1/2 together with a screen for the BRCA1 exon 13 duplication (common in families of British descent) (Puget *et al*, 1999) was used as the minimum testing regime in 89 families. Mutation analysis on the remaining 291 families was performed by a screening strategy that involved the use of a number of different techniques. The protein truncation test (PTT) was performed for exon 11 of BRCA1 and exons 10, 11 and 27 of BRCA2. This test covers 65% of the coding region for each gene and identifies the most common types of pathogenic mutation in BRCA1/2 (Garvin, 1998). Sequence analysis or heteroduplex analysis of exons 2 and 20 of BRCA1 using either polyacrylamide gel electrophoresis or denaturing high-performance liquid chromatography were used to detect nonsense, frameshift and missense mutations within these exons. All samples were screened for the BRCA1 duplication exon 13 mutation and a subset of 125 families from the entire cohort were also screened for rare large genomic rearrangements in BRCA1 using multiplex ligation-dependent probe amplification (MLPA) (Woodward *et al*, 2005).

BRCA1/2 sequence variants of uncertain clinical significance were identified in nine cases and these families were excluded from the analysis; however, families with nondeleterious, common polymorphisms were included in the no-mutation group.

### Statistical methods

All generated scores were analysed using SPSS statistical software V13.0 (SPSS Inc., Chicago, IL, USA). Sensitivity, specificity, positive and negative predictive values, and positive and negative likelihood ratios were calculated for each risk model at the 10% threshold. For the Manchester model, this threshold corresponded with a cutoff score of 10 points for BRCA1 and BRCA2 separately, and a score of 15 points for the combined model. The positive likelihood ratio of a model was calculated as the proportion of individuals with a BRCA1/2 mutation who have a model probability 10% or greater divided by the proportion of individuals without a mutation who have a model probability >10%. Similarly, the negative likelihood ratio was calculated as the ratio of the proportion of individuals with a BRCA1/2 mutation who have a model probability <10% to the proportion of individuals without a mutation who have a model probability <10%. Receiver operator characteristics (ROC) curves were constructed by plotting the sensitivity (or true positive fraction) against 1 minus specificity (or false positive fraction) for all possible values of the mutation probability. The area under the ROC curve (C-statistic) was calculated as a measure of the accuracy of the model for discriminating between mutation carriers and those without a BRCA mutation. It represents the fraction of all probands with identified family mutations that have a detection probability higher than a proband with no mutation identified in the family. The *κ* scores were used to assess the measure of agreement between 100 independent risk calculations from 100 pedigrees chosen at random. In this analysis, a score of 0–0.2 describes slight, 0.2–0.4 fair, 0.4–0.6 moderate, 0.6–0.8 substantial and over 0.8 almost perfect agreement (Koch *et al*, 1977).

## RESULTS

### Proportion of positive BRCA tests varied according to carrier probability

The study cohort included pedigrees from 380 families, of whom 52 (13.7%) carried deleterious mutations in either BRCA1 (34 subjects) or BRCA2 (18 subjects). The mutation frequency was higher (22.5%) in individuals subjected to complete DNA sequencing of BRCA1/2 (20 of 89 individuals) compared with those in whom sequencing had not been performed (11%, 32 of 291 subjects).

A total of 45 different mutations were recorded among the BRCA mutation positive families, whereas duplication of BRCA1 exon 13 occurred in a further five families. The mean carrier probabilities for mutation-positive individuals were 53% using BRCAPRO, 26% for Myriad and 32% using the Penn model. The probabilities for mutation-negative individuals were 24, 14, and 12%, respectively. The mean score for mutation-positive individuals using the Manchester model was 37; the mean score for mutation-negative individuals was 21. All models showed a relationship between the carrier probability and the proportion of positive tests (Table 2).

### Discrimination of BRCA mutation carriers from noncarriers

**Figure 1:**
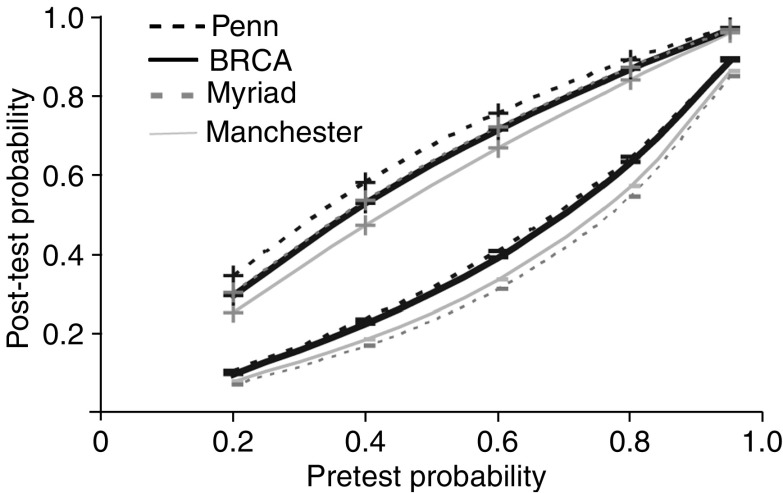
Post-test probability for mutation carrier status is shown for each model using the positive and negative likelihood ratios.

Table 3 shows the sensitivity, specificity, positive and negative predictive values for each risk model at the 10% threshold. The negative predictive value of all four models was remarkably consistent (range=0.93–0.96) in that only 4–6% of families assigned a mutation probability of 10% or less actually carried a mutation in BRCA1 or BRCA2 (Table 3). Given the relatively low prevalence of BRCA mutations in the study population, it is not surprising that the positive predictive value at the 10% threshold was low (range=0.18–0.25). The predictive value improved as the threshold probability was increased; however, 58% of probands assigned an 80% or greater mutation probability using BRCAPRO did not have a mutation detected (Table 2). All probands with a score of 80 or more according to the Manchester model carried a mutation. The likelihood that the model probability was >10% in a mutation carrier was 2.10 (95% CI 1.66–2.67) for Penn, 1.74 (95%CI 1.48–2.04) for Myriad, 1.35 (95% CI 1.19–1.53) for Manchester and 1.68 (95% CI 1.39–2.03) for BRCAPRO. Application of these models, therefore, does not rule in BRCA mutation carrier status. The negative likelihood ratios were consistently greater than 0.1 (BRCAPRO 0.43, Manchester 0.34, Myriad 0.30 and Penn 0.46), which indicated that the models are also unable to rule out mutation carrier status. The post-test probability for mutation carrier status (calculated by multiplying pre-test odds of disease by the positive or negative likelihood ratio) is shown in Figure 1. It is apparent that the estimated pre-test probability of a mutation is not significantly altered by calculating the mutation risk estimate using each of the models. For example, a prediction of a germline mutation for the Penn model would change an individual's pre-test mutation probability from 40 to 58%, whereas using the Manchester model it would increase from 40 to 47%.

**Figure 2:**
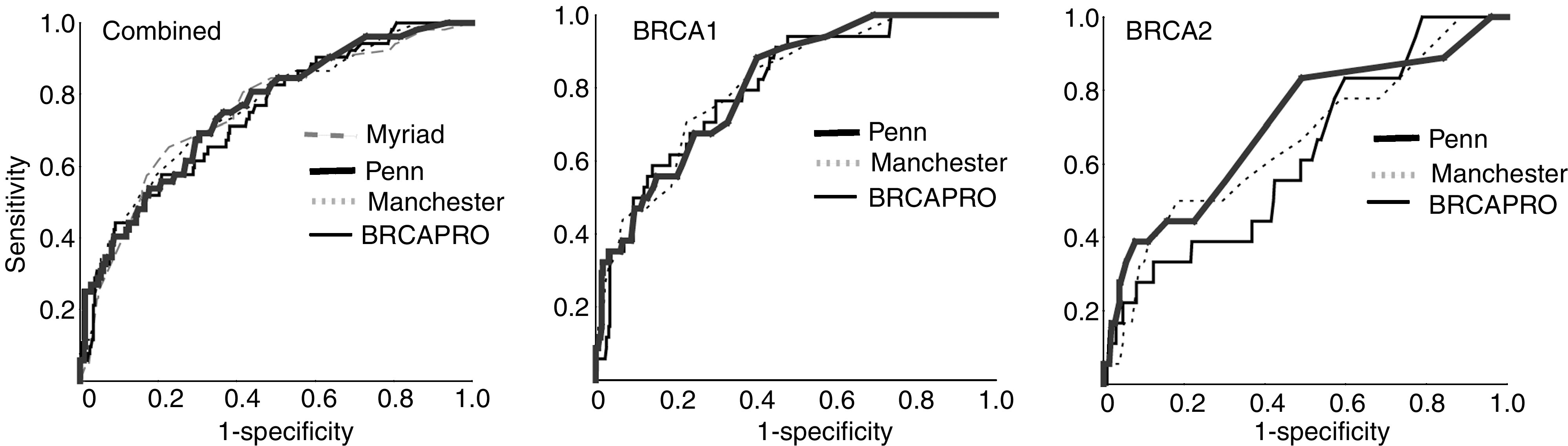
Receiver operator characteristics (ROC) curves for models comprising BRCA1, BRCA2 and the combination. Diagonal segments are produced by ties.

The accuracy of the four models was also compared by examining the areas under the ROC curve (Figure 2 and Table 4). A model that correlates perfectly with BRCA mutation status would have an area under the curve of 1.0 while an area of 0.5 indicates that the model has no discriminatory value. Using the scores for detecting either a BRCA1 or BRCA2 mutation, the area under the curve for each model was close to 0.75 (Table 4). By this measure, the models were equal in their ability to discriminate between mutation carrying and noncarrying probands.

To evaluate the possibility that the mutation-testing strategy influences the performance of the models, we stratified the data according to type of gene sequencing results. Although the frequency of mutation carriers was higher in the group tested by BRCA1/2 sequencing, the models were found to perform similarly for those with and without complete DNA sequencing results. While positive predictive values of models tended to be higher for those with complete DNA sequencing, there were no significant differences in mean model scores or probabilities, sensitivity, specificity, predictive values, likelihood ratios and C-statistics (data not presented).

### Differentiating mutations in BRCA1 and BRCA2

The Manchester, BRCAPRO and Penn models allow separate calculations of the probability of BRCA1 and BRCA2 mutations (Tables 3 and 5). The positive likelihood ratios for BRCA1 mutation carriers were 2.74 (95% CI 1.94–3.88) for Penn, 1.87 (95% CI 1.59–2.21) for Manchester and 2.15 (95% CI 1.72–2.67) for BRCAPRO. The negative likelihood ratios were 0.52, 0.22 and 0.36, respectively. The Penn model could potentially be used to rule in BRCA2 mutations, in that the positive likelihood ratio was 5.93 (95% CI 2.72–12.94) although the negative likelihood ratio was unacceptably high at 0.71 (95% CI 0.51–0.98). The Manchester and BRCAPRO had likelihood ratios around 1, indicating that these models provided no additional information in determining BRCA2 mutation carrier status.

A comparison of the respective ROC curves shows that the Manchester model, BRCAPRO and Penn performed similarly for BRCA1, but that BRCAPRO was the least accurate model for BRCA2 identification (Figure 2, Table 4). The proportions of BRCA1 and 2 mutation carriers at each threshold for the Manchester, BRCAPRO and Penn models are shown in Table 5.

### Validation of risk calculation estimates

Given the inherent ambiguities in interpreting pedigrees, two of the investigators (HK and RWi) specified rules for dealing with various clinical scenarios (see methods and Table 1). At the completion of the study, a *κ* score of mutation-risk estimates using the models was determined for 100 randomly selected pedigrees (25 cases for each risk model). Overall, the *κ* score was 0.82 reflecting excellent agreement between observers when calculating the mutation risk for each proband. The measure of agreement differed between models in that perfect agreement was noted for Penn (*κ*=1.0) and Manchester (*κ*=0.932), whereas only substantial agreement was found for Myriad (*κ*=0.714) and for BRCAPRO (*κ=*0.60). The areas of disagreement in applying the BRCAPRO model were related to clinical judgment on choice of proband, estimation of age of relatives, and inclusion of maternal and paternal relatives.

## DISCUSSION

In this study we used the family histories of a large tested cohort, some with known BRCA germline mutations, to evaluate the clinical effectiveness of four risk prediction models for BRCA mutations. Estimation of pre-test mutation probability is an important clinical issue, particularly in view of the expense, legal implications and other difficulties associated with ambiguous germline BRCA testing results. Germline testing is currently recommended solely on the basis of clinical suspicion, yet even within this group of patients few will harbour a mutation (13% in the current study). While prediction models (Euhus, 2001; Domchek *et al*, 2003) offer an opportunity to improve clinical decision-making, to be effective in clinical practice they must be practical as well as accurate. Our study highlighted certain aspects of the models, which are likely to detract from their effective use in family cancer clinics. The Manchester model (Evans *et al*, 2004) and the Myriad mutation prevalence tables (Myriad, 2004) did not require computer access and were extremely rapid methods of assessing risk. Like most empiric models, the risk estimates relied on the ‘number of cases per family’, yet the definition of a family can be highly subjective. For instance, large pedigrees with many elderly female subjects with breast cancer generated a cumulative score above 10% using the Manchester model. On the other hand, the Myriad tables only allowed inclusion of a maximum of three members of the family, including the patient. Other disconcerting features of the Myriad model were that breast cancers diagnosed above 50 years were ignored, whereas for those diagnosed before 50 years there was no stratification according to the age of diagnosis. Further deficiencies included the equal weighting given to male and female breast cancers and the inability to input bilateral breast cancer or other tumours associated with BRCA1/2 mutation, namely prostate and pancreatic cancers (Lynch *et al*, 1999; Tulinius *et al*, 2002; Liede *et al*, 2004).

Both the Penn model and BRCAPRO required computer access. In the case of BRCAPRO, the time taken to enter family trees was a major impediment to routine use. BRCAPRO only incorporates first- and second-degree relatives and therefore cousins of the proband who are affected with cancer will not be used to generate a probability score unless the counselor changes the proband. This scenario was in part responsible for the low *κ* scores associated with the use of BRCAPRO. The on-line Penn model was a very rapid and efficient mode of calculating not only the individual but also the family’s probability of carrying a BRCA1/2 mutation. The model also provided question prompts with helpful explanatory comments; this feature significantly reduced the chance of disagreement between the independent risk assessors. Unfortunately, the Penn model also restricted questions to three generations, and did not include ovarian cancer only families or mother–daughter ovarian–breast cancer inheritance patterns.

In comparing the accuracy of the models, we found that they had an equal ability to discriminate between mutation carrying and noncarrying probands (C-statistic 0.743–0.759 for combined analysis using the ROC curves) when considering the entire range of possible test thresholds. These results are highly comparable to those of an Italian study of BRCA mutation carriers where risk was assessed using the Myriad tables, BRCAPRO and an old version of the Penn model (Marroni *et al*, 2004). The initial report of the Manchester model claimed superiority over BRCAPRO on the basis of a larger area under the ROC curve (0.772 *vs* 0.596) (Evans *et al*, 2004). We were unable to confirm these findings and note that the area under the curve reported by Evans *et al* for BRCAPRO was considerably lower than that found in the current study as well as a number of other studies (Euhus *et al*, 2002; Marroni *et al*, 2004). The source of these discrepancies may relate to the limitations of comparing a scoring system (Manchester) with probability calculations (BRCAPRO).

Although the area under the ROC curves was comparable between models, this does not mean that they are equally accurate in predicting germline mutation status at the well-accepted 10% probability threshold. Examination of the likelihood ratios allows such a comparison and also provides an assessment of the value of the models in relation to other tools. Penn was the most discriminatory model, in that individuals with BRCA mutation were twice as likely to have a positive prediction as those without a germline mutation. In contrast, the Manchester model had a ratio closer to unity indicating that it is unhelpful in clinical decision-making. In other areas of medicine, likelihood ratios greater than 5 are generally a prerequisite for the adoption of a clinical test or procedure. By way of comparison, the positive likelihood ratio for mammography is 14 (Kerlikowske *et al*, 1995), for bedside cardiac-specific troponin T 6.3 (Antman *et al*, 1995) and for ultrasonography for deep venous thrombosis 47.8 (Wells *et al*, 2000).

The separate BRCA1/2 performance measures for Manchester, BRCAPRO and Penn displayed similar trends to the combined values, with the only exception being the use of Penn for assessment of BRCA2 risk, where the positive likelihood ratio was 5.9. A positive result in Penn for BRCA2 mutation should confidently warrant a mutation screen.

Overall, our study suggests that routine use of Penn, Manchester, BRCAPRO or Myriad for predicting BRCA mutation status in clinical practice is not currently justified. Furthermore, as the performance of the models was not influenced by the type of mutation testing, we should consider other sources of error. Risk models are developed and applied on the basis of pedigrees constructed from clinical histories. Yet, these histories are themselves often inaccurate in that cancer verification is often impossible and age at diagnosis is frequently an estimate. We have shown that even where prespecified criteria are established to address the nuances of various patient scenarios, the expert counselor may still interpret the pedigree in a way that alters the risk estimates. BRCA germline mutation testing represents a further source of significant error. The true prevalence of BRCA mutations is often underestimated because of the limitations of molecular testing (sensitivity of molecular techniques 70%) (Eng *et al*, 2001). Furthermore, models are often derived on the basis of mutation testing results from one uncharacterised individual in a high-risk family. Exclusion of a BRCA mutation in one individual does not necessarily indicate that the family is mutation negative. As uncontrollable factors such as cost, death and unavailability often dictate the choice of individual within a family for mutation testing, it is clear that BRCA mutational status is not a ‘gold standard test’. Given these limitations in developing and applying risk models, we advocate the development of risk prediction models that are less reliant on clinical history.

## Figures and Tables

**Table 1:** Conventions used for interpreting ambiguous pedigrees

**Issue**	**Resolution**	**Applicable model**
Half siblings	Considered full siblings	M, P, B, My
Age of cancer diagnosis unknown	Assigned lowest age possible consistent with current age	M
Bilateral breast cancer	Counted as two different cases	M, My
Bilateral ovarian cancer	Counted as one case	M, P, B, My
Age of living relative unknown	Estimated as 25 years younger than parent or 2 years difference from sibling	B
Age of death not specified	Estimated as 70 years or approximated from age of death of siblings	B
Malignancy age >70 years, age of death unknown	Death estimated as 2 years post diagnosis if cancer is the cause of death	B
Death in infancy	Age of death recorded as 1 year	B
Age of cancer diagnosis unknown	Estimated as 2 years before death for deceased relatives and 2 years younger than current age for living relatives	B

M=Manchester, P=Penn, B=BRCAPRO, My=Myriad.

**Table 2:** The proportion of BRCA mutation carriers according to carrier probability as determined by the four combination models

	**Category of carrier probability (%)^a^**					
	**<10**	**10–20**	**20–40**	**40–60**	**60–80**	**>80**
Manchester	0/19(0.0%)	12/176(6.8%)	22/147(15.0%)	12/31(38.7%)	2/3(66.7%)	4/4(100%)
BRCAPRO	12/190(6.3%)	6/48(12.5%)	4/36(11.1%)	3/24(12.5%)	9/39(27.3%)	18/43(41.9%)
Myriad	8/176(4.6%)	14/115(12.2%)	17/61(27.9%)	13/26(50.0%)	0/2(0.0%)	N/A
Penn	16/232(6.9%)	13/74(17.6%)	8/37(21.6%)	2/12(16.7%)	6/8(75.0%)	7/11(63.6%)

Six families with only ovarian cancer could not be analysed by the Penn model. N/A=not applicable.

a Score, rather than probability, for Manchester model.

**Table 3:** Performance measures for each model at the 10% threshold

	**Proportion of carriers by model probability (%)**		**Test parameters at 10% threshold (95% CI)**			
	**<10**	**⩾10%**	**Sensitivity**	**Specificity**	**PPV**	**NPV**
*Combined*						
Manchester	6/119	46/261	0.89(0.77,0.95)	0.35(0.30,0.40)	0.18(0.14,0.23)	0.95(0.89,0.98)
BRCAPRO	12/190	40/190	0.77(0.64,0.86)	0.54(0.49,0.60)	0.21(0.16,0.27)	0.94(0.89,0.96)
Myriad	8/176	44/204	0.85(0.73,0.92)	0.51(0.46,0.57)	0.22(0.17,0.28)	0.96(0.91,0.98)
Penn	16/232	36/142	0.69(0.56,0.80)	0.67(0.62,0.72)	0.25(0.19,0.33)	0.93(0.89,0.96)
						
*BRCA1*						
Manchester	4/187	30/193	0.88(0.73,0.95)	0.53(0.48,0.58)	0.16(0.11,0.21)	0.98(0.95,0.99)
BRCAPRO	7/225	27/155	0.79(0.63,0.90)	0.63(0.58,0.68)	0.17(0.12,0.24)	0.97(0.94,0.99)
Penn	14/281	20/93	0.58(0.42,0.74)	0.79(0.74,0.83)	0.22(0.14,0.31)	0.95(0.92,0.97)
						
*BRCA2*						
Manchester	6/189	12/191	0.67(0.44,0.84)	0.51(0.45,0.56)	0.06(0.04,0.11)	0.97(0.93,0.99)
BRCAPRO	12/308	6/72	0.33(0.16,0.56)	0.82(0.78,0.85)	0.08(0.04,0.17)	0.96(0.93,0.98)
Penn	12/348	6/26	0.33(0.16,0.56)	0.94(0.92,0.96)	0.23(0.11,0.42)	0.97(0.94,0.98)

PPV=positive predictive value, how likely the patient is to have a mutation given that the model predicts carrier status. NPV= negative predictive value, how likely mutation is not present, given that the model does not predict mutation status.

A Manchester score of 10 for BRCA1 and BRCA2 or a combined score of 15 corresponds with a mutation probability of 10% (Evans *et al*, 2004).

**Table 4:** Area under the ROC curve (C-statistics) for each model

		**95% confidence interval**	
**Test result variable**	**C-statistics**	**Lower bound**	**Upper bound**
*BRCA1 models*			
Manchester	0.808	0.738	0.879
BRCAPRO	0.802	0.731	0.874
Penn	0.808	0.740	0.876
			
*BRCA2 models*			
Manchester	0.660	0.523	0.797
BRCAPRO	0.626	0.500	0.752
Penn	0.703	0.569	0.838
			
*Combination*			
Manchester	0.759	0.688	0.831
BRCAPRO	0.743	0.672	0.814
Myriad	0.753	0.680	0.827
Penn	0.757	0.686	0.827

ROC=receiver operator characteristic.

**Table 5:** The proportion of BRCA1 and BRCA2 mutation carriers according to carrier probability as determined by Manchester, BRCAPRO and Penn

	**Category of carrier probability (%)^a^**					
	**<10**	**10–20**	**20–40**	**40–60**	**60–80**	**>80**
*BRCA1*						
Manchester	4/187(2.1%)	15/150(10.0%)	12/39(30.8%)	3/3(100.0%)	0/1(0.0%)	N/A
BRCAPRO	7/225(3.1%)	4/43(9.3%)	3/37(8.1%)	3/26(11.5%)	6/25(24.0%)	11/24(45.8%)
Penn	14/281(5.0%)	4/42(9.5%)	4/25 (16.0%)	1/7(14.3%)	5/8(62.5%)	6/11(54.6%)
						
*BRCA2*						
Manchester	6/189(3.2%)	6/152(3.9%)	5/37(13.5%)	0/1(0.0%)	1/1(100.0%)	N/A
BRCAPRO	12/308(3.9%)	1/35(2.9%)	2/19(10.5%)	1/9(11.1%)	2/7(28.6%)	0/2(0.0%)
Penn	12/348(3.5%)	5/20(25.0%)	1/6(16.7%)	N/A	N/A	N/A

N/A=not applicable.

a Score, rather than probability, for Manchester model.
